# Patterns of intraspecific morphological variability in soil mites reflect their dispersal ability

**DOI:** 10.1007/s10493-020-00587-y

**Published:** 2021-01-25

**Authors:** Julia Baumann

**Affiliations:** Institute of Biology, Department of Biodiversity and Evolution, University of Graz, Universitätsplatz 2, 8010 Graz, Austria

**Keywords:** Scutacaridae, Heterostigmatina, Geometric morphometrics, Phoresy

## Abstract

The ability to disperse is one of the most important factors influencing the biogeography of species and speciation processes. Highly mobile species have been shown to lack geographic population structures, whereas less mobile species show genetically strongly subdivided populations which are expected to also display at least subtle phenotypic differences. Geometric morphometric methods (GMM) were now used to analyze morphological differences between European populations of a presumed non-phoretic, little mobile mite species in comparison to a highly mobile, phoretic species. The non-phoretic species *Scutacarus carinthiacus* showed a phenotypic population structure, whereas the phoretic species *S. acarorum* displayed homogeneity. These different patterns most probably can be explained by different levels of gene flow due to different dispersal abilities of the two species. GMM proved to be a sensitive tool that is especially recommendable for the analysis of (old) museum material and/or specimens in microscopic slides, which are not suitable for molecular genetic analysis.

**Supplementary Information:**

The online version contains supplementary material available at 10.1007/s10493-020-00587-y.

## Introduction

Dispersal is one of the most important characteristics of all living beings. It can be achieved through active movement or through passive transportation, and dispersal modes are among the most important factors influencing a species’ biogeography (Lomolino et al. 2010) and speciation processes. The phylogeographic patterns of various taxa have been shown to differ clearly between highly mobile species versus species with limited dispersal abilities (e.g., sponge-dwelling shrimps with vs. without swimming larvae, Duffy 1993; winged vs. flightless tenebrionid beetles, Papadopoulou et al. 2009; or various benthic marine invertebrates with high vs. low dispersal potential, Haye et al. 2014). In general, highly mobile species lack geographic population structure, whereas species with limited dispersal show genetically strongly subdivided populations. Morphological characters evolve more slowly than neutral genetic data and thus provide less resolution in analyses of patterns of population differentiation (Miller-Butterworth et al. 2003), but given sufficient time, geographic isolation between populations should also be reflected in subtle morphological differences caused by random Brownian motion (as explained in the glossary of Fišer et al. 2018: “Brownian motion. A model of trait evolution, sometimes called random walk, which assumes neutral and gradual evolution of traits due to genetic drift. Change in mean phenotype is expected to be nondirectional and occurs at a constant rate, whereas variance among species is linearly related to the amount of time since divergence.”). As such subtle morphological differences are not visible by the human eye, the application of adequate morphometric methods to detect them is indispensable.

Mites (Acari) are tiny arachnid organisms that have conquered practically all ecosystems of our planet: they can be found from the deepest depths of the ocean up to mountain heights, they occur in different substrates, and they can even be important pest species affecting plants and animals. Like many other flightless microarthropod taxa, hundreds of mite taxa have huge geographical ranges, which indicates that efficient dispersal pathways must exist– the mode of dispersal, however, is often poorly understood (Schuppenhauer et al. 2019). Given the mites’ minute size, long-distance dispersal by active locomotion can only play a minor role. Anyhow, it seems to be important on a smaller scale, like the immediate colonialization of new habitats (Auger et al. 1999; Lehmitz et al. 2012). Phoresy, the act of using larger and more mobile animals as transport hosts, surely is one of the most effective modes of dispersal for mites as it allows for covering great distances and for targeted reaching of suitable habitats because of host specificity (Binns 1982; Krantz and Walter 2009; Baumann 2018). Usually, mites mount their hosts actively and have morphological adaptations for attachment on the host (like enlarged claws or sucker plates), but phoresy can also happen passively when hosts displace the mites unintentionally, for example when they are transported adhering to birds’ feathers (Lebedeva and Krivolutsky 2003). Other possible mechanisms are dispersal via air currents (anemochory), which has been demonstrated for soil-living oribatids (Lehmitz et al. 2011) and various plant-inhabiting mites (Boykin and Campbell 1984; Jung and Croft 2001; Michalska et al. 2010), or drift on running fresh water (Schuppenhauer et al. 2019) or ocean currents (Pfingstl 2013, 2017; Lindo 2020), both also shown in oribatid mites.

One of the mite families that include species which are considered to be real cosmopolites is the family Scutacaridae (Heterostigmatina, Pygmephoroidea). Scutacaridae inhabit various types of soil, where they feed on fungi, and have been reported from ecosystems of all continents except the Arctic region. Whereas scutacarid females are strongly sclerotized, males and larvae are soft-skinned, can only rarely be extracted from soil samples, and are unknown for the vast majority of species (Jagersbacher-Baumann and Ebermann 2016). The presumed cosmopolitan species have been sampled from locations all around the globe, but comprehensive studies including molecular genetic analyses supporting their species status are lacking. Almost half of all known scutacarid species are associated with other animal taxa such as ants, beetles or mammals, which commonly are used by the females for phoretic dispersal by attaching to their host using large claws on their legs I and thus help the mites to passively disperse over large distances (Baumann 2018). In non-phoretic scutacarid species, the claws on legs I are small or even absent, and their function is unclear. There are also several scutacarid species that, to my knowledge, are not phoretic, but still show a wide distribution (examples can be found in Khaustov 2008). Possible dispersal mechanisms for these species are the same as mentioned above. Whatever the mechanism, it is difficult to imagine a dispersal strategy for scutacarid mites that can be as effective as phoresy.

In the present work, two European scutacarid species represented by specimens mounted in microscopic slides from various European localities were selected to study the differences in morphological patterns between a phoretic and a presumed non-phoretic species. The phoretic species was *Scutacarus acarorum* Goeze, a species using highly mobile bumblebees (genus *Bombus*) for phoresy and thriving in the underground nests of its hosts. *Scutacarus acarorum* is a species with female dimorphism in connection with phoresy: there are phoretic females with large claws and non-phoretic females with tiny claws. When the phoretic females reach a new habitat (that is, a new bumble bee nest), they start to lay eggs and can produce both female morphs. The non-phoretic females stay in the bumble bee nest and are considered to be a kind of ‘energy saving’ variant responsible for rapid reproduction, producing both morphs as well (Baumann 2018). A reduced quality of food in the habitat apparently increases the proportion of phoretic females (Ebermann 1991). European populations of phoretic females of *S. acarorum* have already shown a remarkable morphological homogeneity as revealed by traditional and geometric morphometric analyses (Jagersbacher-Baumann 2015).

Populations of *S. acarorum* were now compared to populations of *Scutacarus carinthiacus* Ebermann, a supposed non-phoretic, soil-inhabiting species with a wide European distribution: it has been reported from Austria, Bosnia, Croatia, Germany, Great Britain, Hungary, Italy and Northern Ireland (Ebermann 1979, 1980, pers. comm.; Khaustov 2008). *Scutacarus carinthiacus* is supposed to be non-phoretic because the species has never been encountered in association with any animal and because it does not possess claws on leg I. Whereas populations of *S. acarorum* showed no phenotypic pattern (Jagersbacher-Baumann 2015), populations of *S. carinthiacus* are now expected to be morphologically more divergent because of reduced dispersal potential resulting in reduced gene flow and neutral evolution of morphological traits due to Brownian motion.

To compare the morphological patterns within the two species, geometric morphometric methods (GMM) based on an already successfully tested set of landmarks were used. In contrast to traditional morphometrics, where lengths, ratios or angles of different body parts are measured and ‘form’ (size and shape) can be analyzed, GMM use landmarks, outlines or surfaces These methods preserve the geometry of shape so that shape can easily be described using graphical representations, and the relationship between size and shape can be explained through allometric trajectories (Adams et al. 2013). GMM have proven to detect very subtle morphological variation (Karanovic et al. 2016), which makes them a valuable tool for the present research question.

## Material and methods

**Fig. 1:**
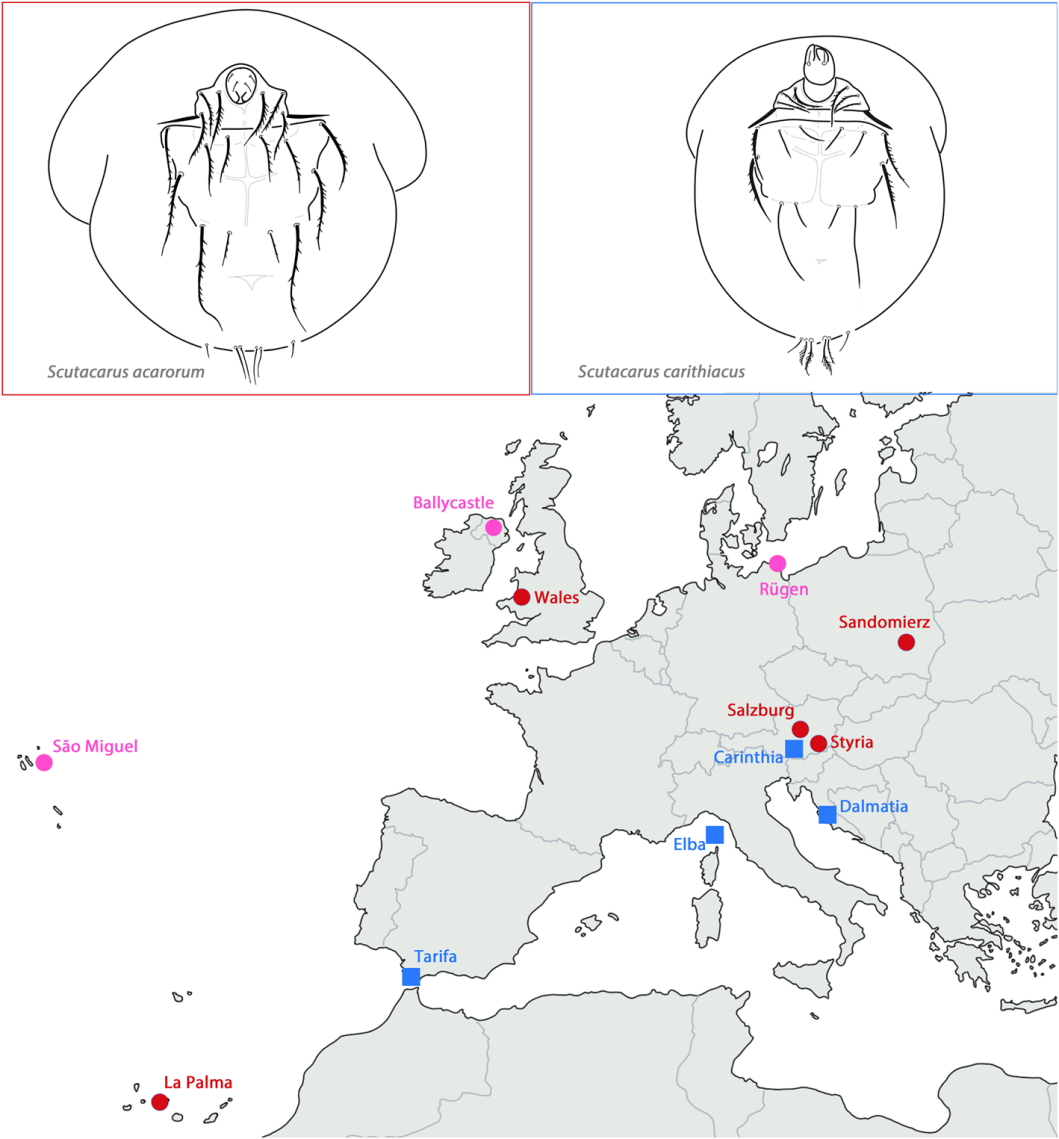
Sample locations of *Scutacarus acarorum* and *S. carinthiacus*. Red dot = *S. acarorum*, blue square = *S. carinthiacus*, pink dot = *S. carinthiacus* single specimens. Simplified drawings of the ventral side, legs omitted, of *S. acarorum* and *S. carinthiacus* are given. (Color figure online)

For the experimental set-up, the optimum case would be a comparison of co-distributed populations of a phoretic and a non-phoretic species from the exact same geographic locations. This was not feasible as the occurrence of scutacarid mite species in soil is virtually unpredictable and their detection depends on more or less random encounters. Because of this, two European species available from different European locations with roughly similar geographic distances between them were selected (Fig. 1, Table 1). Individuals representing the phoretic *S. acarorum* and the supposedly non-phoretic *S. carinthiacus* were available as specimens mounted in microscopic slides in the acarological collection established by E. Ebermann at the Institute of Biology, University of Graz. Additional specimens of *S. carinthiacus* were collected in 2016 by the author. From three locations (Ireland, the island of Rügen in Germany and the Azores), only single specimens of *S. carinthiacus* were available and, thus, they were included in the graphical representation of the results of the Canonical Variates Analysis, but not in further statistical analyses.

**Table 1:** Collection datalocality, sample size (n), collector, year and host/habitat of the studied populations of *Scutacarus acarorum* and *S. carinthiacus*

Locality	n	Collector (of host)	Year	Host/habitat
*Scutacarus acarorum*				
Sandomierz, Poland	10	W. Chmielewski	1984	On *Bombus terrestris*
Wales, Great Britain	10	R. Turk	1985	On *Bombus* sp.
Graz, Styria, Austria	9	G. Kunz	2014	On different *Bombus* species
Thomatal, Salzburg, Austria	9	E. Ebermann	1982	Nest of *B. terrestris*
La Palma, Canary Islands, Spain	6	R. Zarre	2006	On *Bombus* sp.
*Scutacarus carinthiacus*				
Carinthia, Austria	13	E. Ebermann	1995	Mixed forest litter
Tarifa, Spain	12	J. Baumann & F. Ferragut	2016	Litter of *Quercus*
Elba, Italy	9	H. Kaiser	1987	No information available
Dalmatia, Croatia	4	E. Ebermann	1996	Litter of Maquis shrubland
S. Miguel, Azores, Portugal	1	P. Mordan	1999	Moist moss
Rügen, Germany	1	E. Ebermann	2003	Litter of *Fagus*
Ballycastle, Northern Ireland	1	E. Ebermann	2001	Soil

European populations of *S. acarorum* had already been studied through morphometrics (Jagersbacher-Baumann 2015) and were available from various geographic locations. For the present study, three of the populations used in the previous study as well as new specimens from the Canary Islands (La Palma) and Austria (Styria) were chosen in order to cover a geographic range and sample size similar to that of *S. carinthiacus*. As in the previous study, only phoretic females were used because non-phoretic females were only available from Austria.

The two species are easily distinguishable because of morphological features (e.g., ventral setae, Fig. 1) and also because of a clear size difference (average body length of *S. acarorum* analyzed in this study 250 µm versus 170 µm for *S. carinthiacus*). In neither of the two species, discrete morphological differences between the populations could be detected.

**Fig. 2:**
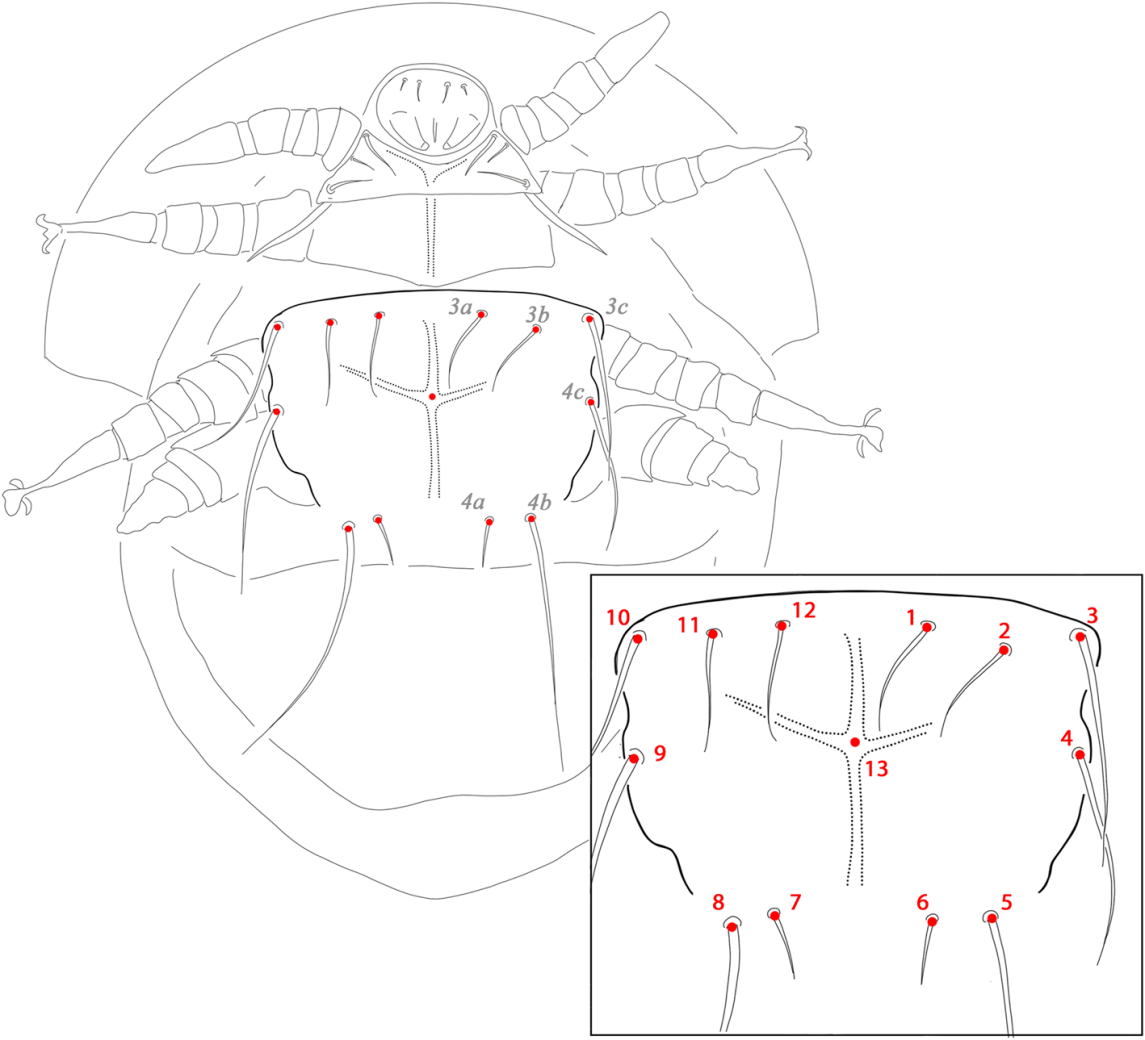
Landmarks on the posterior sternal plate of a stylized scutacarid female mite used for the geometric morphometric analysis

Thirteen landmarks on the sternal plate which had already been successfully used in scutacarid mites (Jagersbacher-Baumann and Ebermann 2012; Jagersbacher-Baumann 2014, 2015) were selected for the geometric morphometric analysis. The landmarks (LMs) consist of the insertion points of the paired setae 3a (LM1 + LM12), 3b (LM 2 + LM11), 3c (LM3 + LM19), 4a (LM6 + LM7), 4b (LM5 + LM8) and 4c (LM4 + LM9) and the crossing between apodeme 4 and the poststernal apodeme (LM13; Fig. 2). Digital photographs of the sternal plates of the studied specimens were taken with a Nikon Eclipse Ni-U compound microscope. A tps file of the photographs was constructed using tpsUtil and the landmarks were digitized using tpsDig2 (Rohlf 2005). The gained coordinates are available in the structure of a ‘.tps’-file (Supplementary file). Further analyses were performed using MorphoJ (Klingenberg 2011) and PAST 3.11 (Hammer et al. 2001). Procrustes superimposition was performed in MorphoJ and the variation within the populations (defined a priori by location) of both species was examined by Canonical Variates Analysis (CVA). For investigating the influence of size, allometric components were identified in MorphoJ and removed by multivariate regression between centroid size and Procrustes coordinates. The power of classification of populations of each species by CVA was evaluated by leave-one-out cross-validation (1000 permutations) in PAST. Mahalanobis and Procrustes distances between the populations were calculated in MorphoJ, and compared between the two species by t-test. Differences in the dispersion between the two species and between the populations within each species were evaluated in R (R Core Team 2020) by permutational analysis of multivariate dispersions with 999 permutations using the function ‘permutest’ in the package *vegan* (Oksanen et al. 2019). Linear geographic distances between populations were measured in GoogleMaps (www.google.at/maps) and the correlation between Mahalanobis distances and geographic distances was evaluated by Mantel test in PAST.

## Results

**Fig. 3:**
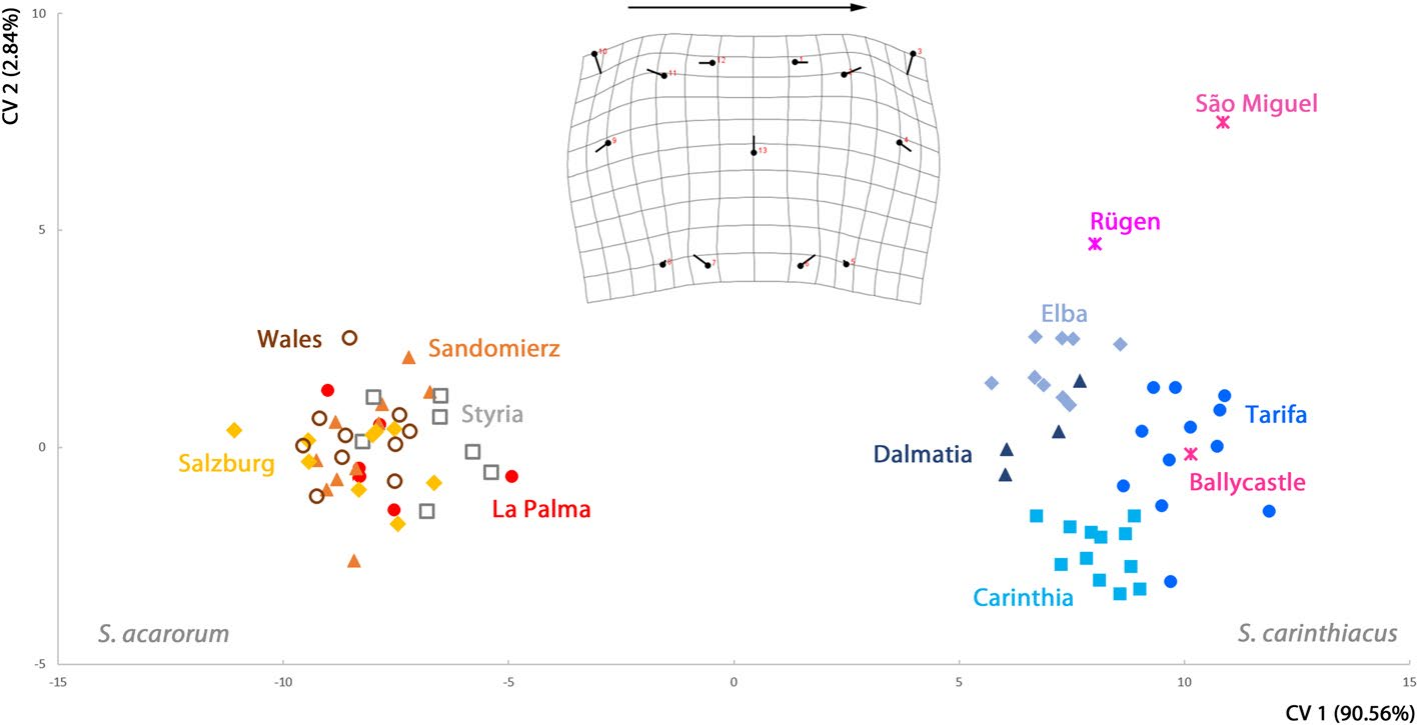
Canonical variates analysis on the shape of the posterior sternal plate of populations of *Scutacarus acarorum* and *S. carinthiacus*, plotting the first two canonical variates. The populations (and single specimens) of both species are color-coded. Transformation grid indicates the shape differences along CV1, scale factor 15. (Color figure online)

*Scutacarus acarorum* and *S. carinthiacus* were clearly separated by CV1 in the CVA on Procrustes coordinates (Fig. 3). In comparison to *S. acarorum*, the posterior sternal plate of *S. carinthiacus* was narrower at level of setae 3c and extended at level of setae 4c. Setae 3a and 3b were located more closely to setae 3c in this species, and setae 4a were in a slightly more anterior position in respect to setae 4b.

Not surprising given the known size difference between *S. acarorum* and *S. carinthiacus*, CV1 was strongly correlated with centroid size (r =  − 0.83). In the CVA performed on both species, allometric effects explained 53.7% of the total variation and the removal of allometric components by regression and subsequent CVA on the remaining residuals resulted in a total overlap of the two species. When the two species were analyzed separately regarding differences between their populations, the allometric effects were weak: they accounted for 2.7% of the variation in *S. acarorum* and 3.2% in *S. carinthiacus*.

The multivariate dispersion (average distance of the specimens to the median of the respective species) did not differ significantly between the two species neither with nor without removal of allometric components. In *S. acarorum*, the multivariate dispersion did not differ significantly between the populations either; in *S. carinthiacus*, the dispersion of the population from Dalmatia was significantly smaller compared to the populations from Elba and from Carinthia in the data before removal of allometric components. After removal of the allometric components, the dispersion of the population from Elba was significantly larger compared to that of the populations from Carinthia and from Tarifa.

**Fig. 4:**
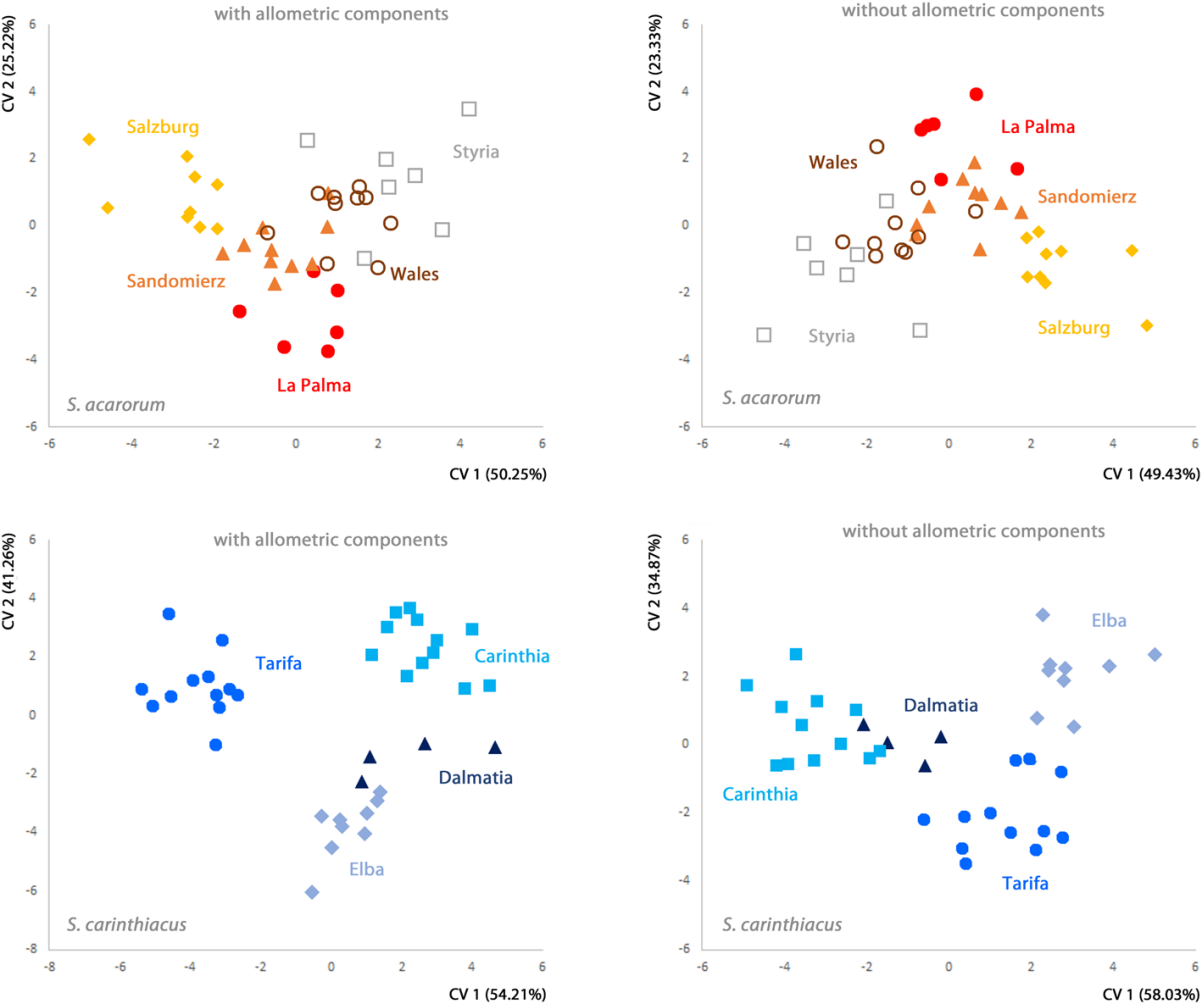
Canonical variates analysis on the shape of the posterior sternal plate of populations of *Scutacarus acarorum* and *S. carinthiacus* analyzed separately (with and without allometric components), plotting the first two canonical variates in each plot. The populations of both species are color-coded. (Color figure online)

In the joint CVA (Fig. 3), the populations of *S. acarorum* overlapped, whereas the populations of *S. carinthiacus* formed distinct clusters. A similar pattern was present when the species were analyzed separately (with as well as without removal of allometric components), although the separation between populations of both species was more pronounced in this case (Fig. 4). Accordingly, the mean of Mahalanobis distances between populations was always significantly larger in *S. carinthiacus* than in *S. acarorum*: in the joint analyses of both species as well as in the separate analyses of each species, and with and without removal of allometric components (Table 2). The mean Procrustes distances also were higher in *S. carinthiacus* than in *S. acarorum*, but they did not differ significantly.

**Table 2:** Mean Mahalanobis and procrustes distances between populations of *Scutacarus acarorum* and *S. carinthiacus*

	*S. acarorum*	*S. carinthiacus*	p	*S. acarorum*	*S. carinthiacus*	p
	Mean Mahalanobis distance between populations			Mean Mahalanobis distance between populations, species analyzed separately		
With allometric effects	3.246	4.331	< 0.01	3.977	5.725	< 0.005
Without allometric effects	3.254	4.033	< 0.05	3.779	5.765	< 0.001
	Mean Procrustes distance between populations			Mean Procrustes distance between populations, species analyzed separately		
With allometric effects	0.022	0.025	n.s	0.023	0.025	n.s
Without allometric effects	0.027	0.037	n.s	0.022	0.025	n.s

P-values are based on t-tests; n.s., p > 0.05

Pairwise differences between populations were highly significant in both species in most cases based on Mahalanobis distances and some pairs of populations also based on Procrustes distances (Table 3). In both species, in some pairings of populations, the distance between them was larger before removal of allometric components and in other pairings the distance was larger afterwards, so no general trend was observable. Only in few cases, the significance of the results changed before and after removal of allometric components.

**Table 3:**
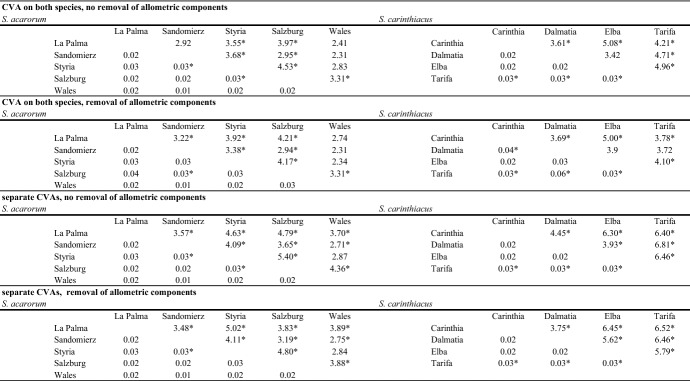
Pairwise distances between populations of *Scutacarus acarorum* and *S. carinthiacus *as gained by CVA

Above each diagonal: Mahalanobis distances; below each diagonal: Procrustes distances

*Indicates highly significant (p < 0.01) differences evaluated by permutation test with 10.000 permutations

More meaningful than the pairwise differences, which were significant in most cases although the differences indeed were very subtle, was the power of classification by CVA: the CVA on both species with allometric components using leave-one-out cross-validation correctly classified 64.1% of *S. carinthiacus* specimens, but only 26.2% of *S. acarorum*. After removal of allometric components, the percentage of correctly classified specimens remained the same in *S. acarorum*, but dropped to 27.0% in *S. carinthiacus*. In the separate analyses of the two species, classification by CVA using leave-one-out cross-validation was also more successful in *S. carinthiacus* than in *S. acarorum*: CVA correctly classified 48.7% (40.5% after removal of allometric components) of specimens in *S. carinthiacus* and only 14.3% (19.1%, respectively) in *S. acarorum*.

No significant correlation between Mahalanobis distances and geographic distance could be detected by Mantel test in neither of the two species, neither when allometric components were present nor when they were removed.

## Discussion

*Scutacarus acarorum* and *S. carinthiacus* cannot only be distinguished from each other because of the already known distinct differences of setae and the general size difference, but also because of a clear shape differences on the posterior sternal plate revealed by GMM. Using the same set of landmarks as in the present study, Jagersbacher-Baumann and Ebermann (2012) detected a clear separation between two population groups from Egypt and from South Africa in the scutacarid mite *Heterodispus foveatus*, caused mainly by differences in the positions of setae 3c, 4a and 4b. The separation revealed by GMM supported the results gained by traditional morphometrics in the respective study, and it was concluded that genetic differences caused by spatial isolation most likely explained the differences.

Analyses of the *acarorum* species-complex, consisting of the four presumed sister species *S. acarorum*, *S. deserticolus*, *S. mendax* and *S. occultatus* (for all except *S. occultatus*, specimens of the phoretic and non-phoretic female morph were available and analyzed separately), showed that morphometric differentiation between the species was more effective using traditional morphometrics than GMM (Jagersbacher-Baumann 2014). However, taking a closer look at the percentages of correctly classified individuals by CVA using leave-one-out cross-validation reveals that 96% of phoretic females and 84% of non-phoretic females could be correctly classified in the data gained by GMM, which are rather satisfying numbers.

These results indicate that the chosen set of landmarks has the power to differentiate between scutacarid species and can even provide sufficient resolution for detecting differences between populations. Future studies will need to test whether also a phylogenetic signal reflects different landmark configurations.

Out of the landmarks used, the ones corresponding to setae 3c and 4c (LMs 3, 4, 9, 10) are located at the very margin of the posterior sternal plate. A different position of these setae presumably indicates that the shape of the posterior sternal plate also has changed, which might influence the inner organization of the mite (e.g., space available for attachment of leg musculature) and thus have a functional implication. Almost all other landmarks indicate insertion points of setae as well (except LM13, which is located at the crossing point between two apodemata), but they are situated within the posterior sternal plate and it is not clear whether a different position of these landmarks could have a strong impact on the mite’s biology. Like the majority of simple setae, all ventral setae are mechanoreceptors (Krantz and Walter 2009), and moderate changes in their position probably do not affect their function much. Thus, a strong selective pressure on the position of the setae does not seem very likely.

The removal of allometric components did not clearly change the encountered patterns between populations in either of the two species. Only in few pairings between populations, the distances between them or the significance of the results changed, but no general trend was observable. This supports the finding that no strong allometry is present within the species: neither in *S. acarorum* nor in *S. carinthiacus* the size of individuals considerably alters the position of the selected landmarks.

As hypothesized, populations of the presumed non-phoretic *S. carinthiacus* showed a clear phenotypic population structure, in contrast to the homogeneous populations of the phoretic *S. acarorum*. The phenotypic homogeneity of Central European populations of *S. acarorum* had already been demonstrated (Jagersbacher-Baumann 2015), and the addition of new populations from Austria and the Canary Islands/ La Palma in the present study did not alter this result. The bumblebee host of the population from La Palma had not been identified to species level by the collector, but as *S. acarorum* prefers large-bodied species as host and because no other bumble bee species has been reported from the Canary Islands so far (Arechavaleta et al. 2010), it is safe to assume that it was *Bombus terrestris canariensis*. Because of geographic isolation, this subspecies of *B. terrestris* is genetically and also morphologically well separated from European mainland populations, which in turn display homogeneity (Estoup et al. 1996; Widmer et al. 1998; Lecocq et al. 2013). In contrast to their host, Canarian *S. acarorum* mites are not morphologically distinguishable from their mainland conspecifics.

The phenotypic homogeneity within the *S. acarorum* populations from continental Europe can be explained by a bottleneck event in the last ice age, a rather recent redistribution at the beginning of the last warm period and a well-maintained gene flow because of the high mobility of the bumblebee hosts (Estoup et al. 1996; Widmer et al. 1998; Jagersbacher-Baumann 2015). A similar result was obtained for *Imparipes burgeri*, a scutacarid mite that is phoretic on a variety of Central European Hymenoptera (most of all on Halictidae). In a traditional morphometric study of populations from Austria, Germany and Belgium associated with a total of 45 host species, Ebermann et al. (2013) also revealed morphological homogeneity of the *I. burgeri* populations. It is more complicated to find an explanation for the lack of morphological differences between the mites from La Palma and from continental Europe. The earlier study on *S. acarorum* also included a population from New York (USA), and not even this population showed clear morphometric differences from the European populations (Jagersbacher-Baumann 2015). These results indicate that *S. acarorum* has a very conserved phenotype not only because of well-maintained gene flow, but also because of morphological stasis. Such a morphological stasis has also been reported, for example, for littoral oribatid mites, where it is supposed to be the result of stabilizing selection due to the extreme habitat of the littoral zone (Pfingstl et al. 2019). Morphological stasis in general is considered to be the result of low-standing genetic variation, developmental constraints on the morphospace, and/or a relatively constant ecology of a taxon (Struck et al. 2018). The microclimate inside belowground bumblebee nests is favorable for mites because of an abundance of stored food reserves and debris (Baumann 2018); moreover, it is considered to be little variable even across geographical localities (Haas et al. 2019), and these stable conditions could explain the morphological stasis of *S. acarorum*. Another important stabilizing factor leading to the conserved phenotype might be the phoretic lifestyle. The results of other studies on Scutacaridae (Ebermann 1991; Jagersbacher-Baumann 2014) also support the idea that phoresy may constrain the morphospace: the respective studies indicate that in dimorphic scutacarid species (such as *S. acarorum*), the intraspecific morphometric variability of non-phoretic females is higher than that of phoretic females. The more conserved phenotype of the phoretic females might be necessary for the successful performance of phoresy. Unfortunately, non-phoretic females of *S. acarorum* were not available from different geographic locations to test whether their morphological pattern mirrors that of their phoretic conspecifics. Similar host-associated adaptive pressures have also been reported for other ectosymbionts such as feather mites (Stefan et al. 2018).

In contrast to *S. acarorum*, the populations of *S. carinthiacus* showed clearer phenotypic differences, which suggests that there is no or only very limited gene flow between them. At present, it is not possible to determine whether the subtle morphometric disparity is due to neutral evolution or due to adaptation to ecologically different (micro)habitats between geographic locations. In any case, there seem to be no constraints leading to morphological stasis like in *S. acarorum*.

No phenogeographical pattern linking the phenotypic differences with geography was observable: for example, the Mahalanobis distances between the populations from Carinthia and Elba were higher than the Mahalanobis distances between the populations from Carinthia and Tarifa, although the linear geographic distance between the latter is considerably higher (520 vs. 2005 km). Geographic vicinity alone apparently is not sufficient to reconstruct the phenotypic (and probably also phylogenetic) relationships or colonization routes of *S. carinthiacus*. Our knowledge about the dispersal mechanisms of small flightless arthropods that are non-phoretic is still very limited, and they have never been studied in non-phoretic Scutacaridae. As mentioned before, *S. carinthiacus* is considered to be non-phoretic because it is a species lacking claws on legs I (Baumann 2018). However, a recent discovery demonstrated that there are also scutacarid species that perform phoresy although they do not possess claws on leg I and instead use large pulvilli for attachment on their termite host (Baumann et al. 2018); this finding renders the previous strict division of phoretic and non-phoretic scutacarid species uncertain. The results of the present study still support the assumption of a reduced capability to cross geographic barriers in *S. carinthiacus*; occasional dispersal through phoresy might have happened in the past, explaining the wide geographic range of the species. Phoresy still might take place occasionally at the present, but since *S. carinthiacus* never has been encountered in association with potential hosts, it seems not very likely.

At least some of the geographically and morphometrically separated populations of *S. carinthiacus* studied here could represent ‘cryptic’ species. The concept of cryptic species refers to species that are morphologically indistinguishable, but show sufficient genetic differences so that it seems justified to establish them as separate species, and such species are being discovered in virtually all animal taxa (e.g., Fišer et al. 2018; Korshunova et al. 2019). Cryptic speciation is a strongly discussed topic nowadays and is controversial because of different species concepts, but also because of different approaches on how to define morphological disparity. More thorough studies conducted after molecular genetic analyses (for example, on fine-scale morphology, behavior, chemical properties) usually support the new species status (Skoracka et al. 2015), which indicates that ‘cryptic’ species often only are an example of inadequate taxonomy. In mites, several studies demonstrated that genetically well separated species did not show differences in distinct morphological characters and only differed slightly in morphometric analyses (e.g., Knee et al. 2012; Pfingstl et al. 2019; Schäffer et al. 2019). The morphometric differences revealed in *S. carinthiacus* thus point to the possible existence of a species complex, even more so as phenotypic variation analyzed by GMM can be as sensitive as molecular data (Karanovic et al. 2016). However, since the present study had another objective and was only based on landmarks on the posterior sternal shield, the identification of new species is not justified yet.

## Conclusion

The present study showed that geographically separated populations of two scutacarid species with presumed different dispersal abilities display different phenotypic patterns. Whereas distinct diagnostic morphological characters did not differ between the populations of either species, clear morphometric differences were detected in one of the species via GMM. The differences in the phenotypic patterns of *S. acarorum* and *S. carinthiacus* can probably be explained by different levels of gene flow between the populations of the two species caused by differing dispersal abilities.

Mite material in long-established acarological collections often is only available in the form of microscopic slides or had been stored inadequately (e.g., in low-concentration ethanol at room temperature) so that molecular genetic analyses are not feasible. GMM exceed the possibilities of traditional morphological analyses and can thus be recommended as a powerful and sensitive tool for the study of such material (e.g., analysis of morphological variation, detection of cryptic diversity, or reconstruction of morphology-based phylogenies).

## Supplementary Information

Below is the link to the electronic supplementary material.

Supplementary file1 (XLSX 32 KB)
